# Cognitive-Enhancing Effects of Polygalasaponin Hydrolysate in A*β*_25–35_-Induced Amnesic Mice

**DOI:** 10.1155/2011/839720

**Published:** 2011-03-06

**Authors:** Shu ping Xu, Yan yan Yang, Dan Xue, Jin xiu Liu, Xin min Liu, Tai-Ping Fan, Rui le Pan, PengTao Li

**Affiliations:** ^1^Research Center of Pharmacology and Toxicology, Institute of Medicinal Plant Development, Chinese Academy of Medical Sciences and Peking Union Medical College, Beijing 100193, China; ^2^Dongzhimen Hospital, Beijing University of Chinese Medicine, Beijing 100700, China; ^3^Angiogenesis & Chinese Medicine Laboratory, Department of Pharmacology, University of Cambridge, Cambridge CB2 1PD, UK

## Abstract

Polygalasaponins are the major active constituents of *Polygala tenuifolia* exhibiting antiamnesic activity, but their applications are limited due to their toxicities. Evidence showed that the toxicities can be attenuated by hydrolysis. Herein, effects of a hydrolysate of polygalasaponins (HPS) on cognitive impairment induced by A*β*_25−35_ were assessed by Morris water maze and step-through passive avoidance tests. The impaired spatial reference memory was improved by HPS (50 and 100 mg/kg). In the acquisition trial of step-through test, HPS (50 and 100 mg/kg) increased the latency into the dark chamber and decreased the error frequency significantly (*P* < .05). However, no significant change was observed during the retention trial. Additionally, HPS increased the corresponding SOD activities (62.34%, 22.09%) and decreased MDA levels (28.21%, 32.35%) in both cortex and hippocampus as compared to model animals. These results show that HPS may be a useful treatment against amnesia probably via its antioxidant properties.

## 1. Introduction

With global increases in population and life expectancy, Alzheimer's disease (AD), a neurodegenerative disorder characterized by progressive memory loss and cognitive deterioration, has become a major health problem [1]. Despite the fact that more than 35 million people will be suffering from AD by 2010, there are only few treatment options available [2]. Thus a search for novel pharmacotherapies especially from traditional medicines for AD has recently been the focus of many researchers.

*Polygala tenuifolia *(called* “Yuanzhi” *in Chinese) has been widely used as a memory enhancer for people in Asian countries for many years [3–5], and it is also included in many traditional prescriptions used for treatment of amnesia and dementia [6–10]. Recently, preclinical trials showed that ethanol extracts of *P. tenuifolia *possess memory-enhancing effects in healthy [11] and elderly volunteers [12]. There is a growing support for improvement of memory function in many animal models with learning and memory impairment induced chemically or physically, for example, by scopolamine [13–15], KCN [13], stress [16], and hypoxia [17]. Additionally, it is also effective on aging [14, 18] and on senescence-accelerated prone (SAMP8) mice [19]. This cognitive improvement by *P. tenuifolia* has been associated with polygalasaponins as its main constituents [13–15]. However, it has been reported that the polygala saponin fraction was highly toxic in animals [20, 21], and even caused death to SD rats (0.5 mg/kg, i.p.) [22] and ICR mice (100 mg/kg, p.o.) [13], which seriously limits the application and development of an effective agent from *P. tenuifolia *for AD treatment. Some studies on the correlation between the molecular structures and toxic activities of the components suggested that removal of the oligosaccharides at C-28 by hydrolysis will attenuate or markedly abrogate the toxic effects [23]. 

But whether the hydrolysate of polygalasaponins (HPS) has an antiamnesic effect has not been investigated. In the present study, its effect on cognitive improvement in amnesia C57BL/6J mice was undertaken. Amnesia was induced by A*β*_25–35_ injection into the hippocampus. Following two behavioral tests (Morris water maze and step-through passive avoidance test), potential mechanisms were investigated.

## 2. Materials and Methods

### 2.1. Preparation of HPS

Air-dried root barks of* P. tenuifolia Willd* were purchased from the Company of Chinese Materia Medica in Beijing and identified by Professor Ruile Pan from the Institute of Medicinal Plant Development, Chinese Academy of Medical Sciences (Beijing, China). A voucher specimen (no. 20090815) has been deposited in the Herbarium of the institute. The dried roots (1 kg) were cut into small pieces and extracted exhaustively with boiling water for 1 h four times. The filtered liquid was loaded to a column of macroporous resin D101 (4 kg) and eluted with water, 30% ethanol, 95% ethanol successively. The 95% eluent (enriched in saponins) was concentrated by evaporation of the ethanol, and subsequently hydrolyzed 4 hours at pH 14 at 100°C. After cooling, it was passed through a column of macroporous resin D101 (4 kg) and eluted with water and then 95% ethanol. The 95% ethanol extract was evaporated under vacuum to yield a pale yellow residue (HPS 25 g) and stored in a refrigerator.

### 2.2. Determination of HPS by HPLC

HPS was analyzed by HPLC using Waters chromatographic equipment with a control 600E pump, 2487 UV detectors and Empower software. HPLC conditions are as follows: a LiChroCART C18 Column (5 *μ*m, 250 mm × 4.6 mm, Merck, Darmstadt, Germany); detection wavelength 210 nm; gradient elution using an A eluent (MeOH) and a B eluent (0.1%, v/v, H_3_PO_4_ in water) with the following linear gradient combinations. 0 min: 30% A, 60 min: 90% A. Total run time was 60 min. Flow rate was 1 mL/min. The contents of tenuifolin, fallax saponin A, 3, 4, 5-trimethoxy cinnamic acid, and p-methoxycinnamic acid were 289.5, 197.2, 247.1, and 770 mg/g, respectively.

### 2.3. Drugs and Reagents

Amyloid beta-Protein fragment 25–35 (A*β*_25–35_) and pentobarbital sodium were from Sigma, galantamine hydrobromide from the National Institute for the Control of Pharmaceutical and Biological Products, superoxide dismutase (SOD) and malondialdehyde (MDA) kit from Nanjing Jiancheng Bioengineering Institute. All the other reagents were analytical grade from Sigma.

### 2.4. Fibrillar A*β*_25–35_ Peptide Preparation

A*β*_25–35_ peptide was dissolved in sterile saline and incubated at 37°C for 7 days to allow fibril formation as described previously [24]. The fibril-like structures and globular aggregates were confirmed microscopically.

### 2.5. Animals

Male C57BL/6J mice (26–30 g) were purchased from the Laboratory Animal Institute of the Chinese Academy of Medical Science (qualified no.: SCXK 2004-0001, Beijing, China). They were housed in groups of 5 animals per cage under a 12 : 12 h light-dark cycle at constant temperature (23°C ± 2°C) and humidity (50% ± 10%). The animals had free access to standard chow diet and sterilized drinking water in the SPF animal house. All animal experiments were conducted in compliance with the “Guide for the Care and Use of Laboratory Animals” of the Institute of Medicinal Plant Development (Chinese Academy of Medical Science and Peking Union Medical College).

### 2.6. Apparatus

Open field, Morris water maze, and step-through passive avoidance computer-aided controlling systems were all developed by the Institute of Medicinal Plant Development, Chinese Academy of Medical Sciences, and the Chinese Astronaut Center (Beijing, China).

### 2.7. In Vivo Experimental Procedure

**Figure 1:**
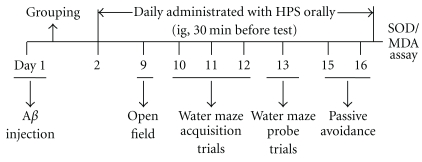
Experimental procedure.

After 7 days of habituation, mice were subjected to unilateral hippocampus injection of vehicle (saline) or A*β*_25–35_ (1 mg/mL). 24 h later, the animals received orally either water (control, sham, and model) or various concentrations of HPS (50 and 100 mg/kg) and galantamine (3 mg/kg) for a period of 15 days. After surgery, the mice were sequentially tested in open-field on day 9, Morris water maze (day 10 to 13), and step-through passive avoidance on day 15 and 16. Then, the mice were decapitated, and the cortex and hippocampus were dissected quickly on ice for detection of SOD activities and MDA contents. The procedure is presented in Figure 1.

### 2.8. Surgical Procedure

**Figure 2:**
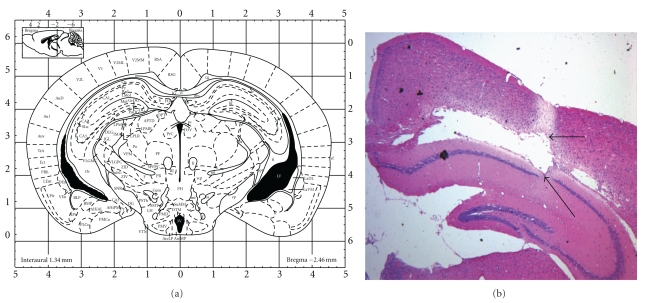
Schema of a coronal (a) section of the mouse brain showing the microinjection sites in the hippocampus. (b) An image of the hippocampus stained with hematoxylin and eosin stain (H&E stain). The black arrows represent the needle path and hippocampal lesions.

Animals were anesthetized (*ip*) by pentobarbital sodium (0.4%, 0.2 mL/10 g) and positioned in a stereotaxic frame (Benchmark Company, stereotaxic 463701, USA) for unilateral intracranial injection (right side) of aggregated A*β*_25–35_ (6 *μ*L, 1 mg/mL) in the CA1 area of the dorsal hippocampus using the coordinates derived from the atlas of Paxinos and Franklin [25]: 2.46 mm posterior to bregma, 2.0 mm lateral to the sagittal suture. These injections (sterile saline or A*β*_25–35_) were made 2.0 mm beneath the surface of the skull using a microsyringe (10 *μ*L) equipped with a 26S-gauge needle at an infusion rate of 1 *μ*L/min for 6 mins. To avoid effusion, the needle was removed after an additional 3 min. After the injection, the scalp was sutured and the animals were allowed to recover from anesthesia. After the surgical procedures the mice were randomly divided into six groups (*n* = 10 per group): control, sham control, A*β*_25–35_ model, A*β*_25–35_ + positive group (galantamine 3 mg/kg/d, ig), and A*β*_25–35_ + HPS-treated groups (50 and 100 mg/kg/d, ig). The stereotaxic coordinates employed are shown in Figure 2.

### 2.9. Open-Field Test

To verify the effects of the treatment on locomotor activities, the animals were evaluated automatically using an open field computer-aided controlling system as described earlier [26–28]. The apparatus consists of four metal tanks (diameter 30 cm, height 40 cm) with a video camera fixed at the top. Experiments were performed in a quiet room, and the apparatus was illuminated by a light source of 120 Lux on the ceiling. Thirty minutes after drug administration, each mouse was placed at the center of the metal tank and allowed to explore freely for 5 min and the distance travelled in 10 min, which is an index of locomotor activities, was calculated using software. Four mice were tested simultaneously.

### 2.10. Morris Water Maze Test

#### 2.10.1. Apparatus

 The apparatus is a circular water pool (100 cm in diameter and 40 cm in height) with constant clues external to the maze (e.g., bench, pictures, lamps, etc.) for spatial orientation of the mice. The water was made opaque by adding skimmed milk to prevent animals from seeing the submerged platform. The water temperature was kept at 24–26°C during the experiment. An invisible platform (6 cm diameter, 15 cm high) providing the only escape from water was placed 1.5 cm below the water surface. The pool was divided into four quadrants by a computerized tracking and image analyzer system. Two principal axes of the maze intersect perpendicularly to one another to create an imaginary “+”. The end of each line demarcates one of the four cardinal points: north (N), south (S), west (W), and east (E).

#### 2.10.2. Testing Procedure

On day 10 after surgery, the Morris water maze paradigm was used to assess the spatial reference memory which reflects the long-term memory ability. This behavioral task consists of an acquisition phase and a probe trial. The protocol was similar to that described by Morris [29] with minor modifications [26].

In the acquisition phase, mice were submitted to daily sessions of four trials per day for 3 days to find the submerged platform that was located in the center of the SE quadrant of the pool and remained at the same position throughout the whole experiment. For each trial, the mouse was placed for 15 sec on the platform for learning, then it was gently released into the pool facing the wall. Four different release points (NE, SE, SW, and NW) were varied semirandomly for each trial. Animals were given a maximum of 60 sec to find the platform. If the mouse failed to find the platform within 60 sec, it was gently guided to the platform and stayed there for 10 sec and its escape latency was recorded as 60 sec. If an animal found the platform within 60 sec, it was allowed to remain there for 10 sec, and was then placed into a cage until the next trial. After completion of daily training, the animals were returned to their home cages for rest. Escape rate, escape latency, cumulative distance, and swimming speed were collected for subsequent analysis. 

24 h later, each mouse was subjected to the retention test (probe trial) in which the platform was removed. The mice were released into the water on approximately the opposite side of the SE quadrant. They were allowed to swim freely for 60 s. The number of crossings over the position at which the platform had been located, and the swimming time and distance spent in the quadrant of the former platform position were recorded as measures for spatial memory.

### 2.11. Passive Avoidance Test

A step-through-type passive avoidance test was carried out to evaluate the effect of HPS on learning and memory. The apparatus and procedure were the same as describe by Xue et al. [30]. The shuttle box (60 × 50 × 80 cm) consisted of two compartments of equal size (20 × 12 × 60 cm). Each compartment was divided into a light and a dark chamber by a guillotine door (3 × 4 cm). The light chamber was equipped with an illuminator. All the mice were habituated to the light and dark chamber 3 times for 3 minutes until they entered the dark compartment within 15 sec (training trial). The mice that did not enter the dark compartment within 15 sec after the 3 habituations would be rejected. The test consisted of acquisition and retention sessions carried out 24 and 48 hr, respectively, after the training trial. For the acquisition trial, the mice were placed in the illuminated chamber opposite the guillotine door and allowed to move freely. When all four paws were on the grid floor of the dark compartment, a scrambled constant-current foot shock (constant voltage 50 V) was delivered to the grid. The number of times the mice stepped into the dark chamber, the time spent in the dark chamber, and latency to move into the dark chamber within 5 min were recorded automatically by computer. 24 hr after the acquisition trial, the mice were again placed in the light chamber for the retention trial. If a mouse did not enter the dark chamber within 300 sec, the latency was recorded as the cutoff time of 300 sec. Mice were removed manually from the light chamber when the cutoff time was reached.

### 2.12. Preparation of Brain Tissue and Homogenates

After the final behavior test, all mice were sacrificed by decapitation. The brains were removed immediately. For biochemical studies, the cerebral cortex and hippocampus were dissected on ice cubes and homogenized in 10 volumes of cold saline using a glass homogenizer. The homogenates were centrifuged at 2000 rpm for 10 min at 4°C, and the supernatants were used for biochemical determinations.

### 2.13. Determination of SOD Activity and MDA Content in Cortex and Hippocampus

Total SOD activities and MDA levels were measured using commercial kits (Nanjing Jiancheng Bioengineering Institute, Nanjing City, China). All procedures complied with the manufacturer's instructions. The assay of total SOD was based on its ability to inhibit the oxidation of oxymin by the xanthine-xanthine oxidase system [31]. The activities were quantified by measuring the amount of hydroxylamine nitrite produced by the oxidation of oxymin. One unit (U) of SOD was defined as the amount that reduced the absorbance at 550 nm by 50%, and data were expressed as units per mg protein. MDA contents were determined by the thiobarbituric acid reaction (TBAR). A stable chromophoric product formed by reaction with thiobarbituric acid (TBA) can be measured at the wavelength of 532 nm [32]. MDA levels were expressed as nmoles per mg protein. The protein concentration in the brain supernatant was estimated using a bicinchoninic acid (BCA) kit with bovine serum albumin as a standard at the concentration of 2 mg/mL.

### 2.14. Data Analysis

All tests were analyzed using the SPSS statistic 17.0 (SPSS Inc., Illinois, Chicago, USA). Data were expressed as mean ± S.E.M (*n* equals the number of mice included in each analysis). One-sample Kolmogorov-Smirnov test was used to test data distribution. If the data were normally distributed, the statistical evaluation of the results was carried out using one-way analysis of variance (ANOVA). Following significant ANOVAs, multiple *post hoc* comparisons were performed using the LSD test (homogeneous variances) or Games-Howell test (nonhomogeneous variances). When the data distribution was not normal, the Kruskal-Wallis *H* test was used to compare the differences between groups, and differences between pairs of medians were analyzed using the Mann-Whitney *U*-test. For the acquisition trails of Morris water maze, mean escape latencies and escape rates were recorded for each day (four trials per day), and the data were analyzed with repeated measures and a multivariate analysis of variance (ANOVA) process of the general linear model. The symmetry of covariance matrices in the analyses was tested using Mauchly's test of sphericity. If *P* < .05, *F*-ratios were calculated using the Greenhouse-Geisser correction. The accepted level of significance for the tests was *P* < .05. Graphpad Prism version 5.00 for Windows (GraphPad Software, San Diego California, USA) was used for plotting.

## 3. Results

### 3.1. Effect of HPS on Locomotor Activities in the Open Field Test

**Figure 3:**
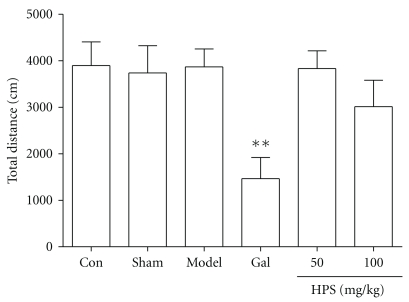
Effect of HPS on locomotor activities of mice. Data represent the means ± SEM. *n* = 8–10 in each group. Gal represents galantamine 3 mg/kg group. The total distance travelled by mice was measured after repeated administrations of HPS (50, 100 mg/kg, p.o.) for 8 days. **P < .01 compared with model group.

As shown in Figure 3, no significant effect of HPS (50 and 100 mg/kg, ig, 8 days) on locomotor activities was observed in the novel environment, although the total distance was slightly reduced in the HPS (100 mg/kg) group compared with the A*β*_25–35_-injected group (Games-Howell test, *P* = .865). Galantamine 3 mg/kg reduced the total distance significantly compared with model groups (Games-Howell test, *P* = .007).

### 3.2. Effect of HPS on Spatial Reference Memory Deficits Induced by A*β*_25–35_ in MWM

#### 3.2.1. Acquisition Trails

**Figure 4:**
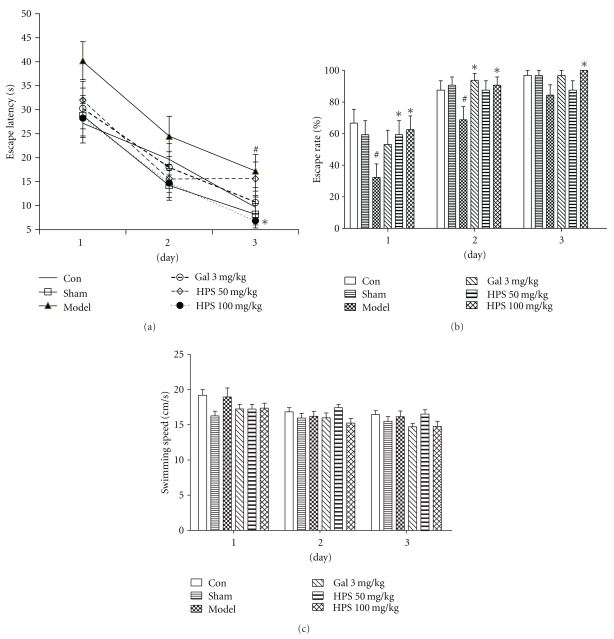
Effect of HPS on the acquisition of spatial reference memory in MWM task on amnesic mice induced by intra-hippocampus A*β*_25–35_ microinjection. Training trials were carried out on day 9 after treatments. (a) Escape latency, (b) mean speed, and (c) escape rate of mice to the hidden platform across the 3 days of training trials (4 trials per day). Gal 3 mg/kg represents galantamine 3 mg/kg group. Values represent mean ± S.E.M, *n* = 8–10 in each group. ^#^ *P* < .05, ^##^ *P* < .01 compared with sham-operation group; *P < .05, **P < .01 compared with model group.

Two-way repeated measures ANOVA (day × group) revealed that the group had a significant effect on the escape latency (*F*_1,5_ = 4.63, *P* = .002) and escape rate (*F*_1,5_ = 5.24, *P* = .001). Also a significant day effect on the escape latency (*F*_2,41_ = 49.48,  *P* < .001) and escape rate (*F*_2,41_ = 62.38, *P* < .001) was observed, which indicated that mice improved over the course of training trails in all groups. However, there was no significant interaction between these two factors (day × group) in escape latency (*F*_10,84_ = 0.48, *P* = .899) and escape rate (*F*_10,84_ = 0.812, *P* = .618) during 3 days training. Subsequent comparisons further suggested that no difference was observed between the control and sham-operated groups on escape latency (day 1, *P* = .805; day 2, *P* = .129; day 3, *P* = .402) and escape rate (day 1, *P* = .556; day 2, *P* = .691; day 3, *P* = .982). The mice injected with A*β*_25–35_ took a longer time to locate the platform than did mice in the sham group (Figure 4(a)), but it became significant only on day 3 (day 1, *P* = .073; day 2, *P* = .175; day 3, *P* = .03). Interestingly, when compared by the parameter escape rate (Figure 4(b)), significant differences between model and sham-operated groups were observed on day 1 and day 2 (day 1, *P* = .032; day 2, *P* = .031), and no significant differences on day 3 (*P* = .096) when over 80% mice could find the platform. 

HPS (100 mg/kg) attenuated the spatial learning deficits observed following A*β*_25–35_ microinjection (10 days later), as indicated by a reduction of the escape latency (day 1, *P* = .013; day 2, *P* = .235; day 3, *P* = .032), and an increase of escape rate (day 1, *P* = .017; day 2, *P* = .031; day 3, *P* = .021) in comparison to the model group. HPS (50 mg/kg) group showed a trend, but it was not statistically significant of decreasing the escape latency (day 1, *P* = .136; day 2, *P* = .582; day 3, *P* = .347): it significantly increased the escape rate only on day 1 (day 1, *P* = .032; day 2, *P* = .072; day 3, *P* = .721). Similarly, the treatment with galantamine (3 mg/kg), a positive agent, also slightly increased the escape rate (day 1, *P* = .097; day 2, *P* = .011; day 3, *P* = .089), and reduced the escape latency (day 1, *P* = .108; day 2, *P* = .456; day 3, *P* = .151) during 3 days, at least at the dose tested. There were no significant differences in the swimming speeds among all the groups (Figure 4(c)).

#### 3.2.2. Probe Trail

**Figure 5:**
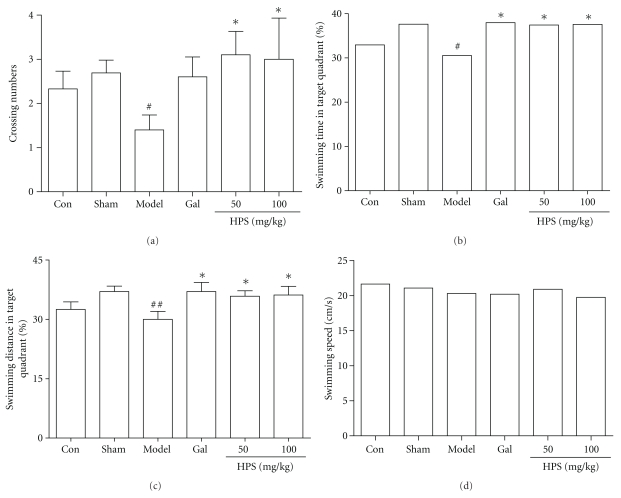
Effect of HPS on memory retention in probe trial of MWM task on amnesic mice induced by intrahippocampal A*β*_25–35_ microinjection. The probe trial was performed 24 h after the acquisition phase. (a) Crossing numbers, (b) swimming time in target quadrant, (c) swimming distance in target quadrant, and (d) swimming speed. Gal represents galantamine 3 mg/kg group. Values represent mean ± S.E.M (*n* = 8–10 in each group). ^#^ *P* < .05, ^##^ *P* < .01 compared with sham operation group. *P < .05 compared with model group.

As can be seen from Figure 5(a), the number of platform site crossings was significantly lower in the model mice than sham-operated mice (*P* < .05). Galantamine (3 mg/kg) slightly increased the crossing numbers but without statistically significant difference. By contrast, HPS (50 and 100 mg/kg) increased the crossing numbers markedly (*P* < .05), and the shorter swimming time in the target quadrant induced by A*β*_25–35_ was significantly reversed by HPS (50 and 100 mg/kg) or galantamine (3 mg/kg) (Figure 5(b), *P* < .05). A similar pattern was seen for the swimming distance in the platform quadrant (Figure 5(c)). However, no significant differences were observed in swimming speeds in the probe trail (Figure 5(d)).

### 3.3. Effect of HPS on Cognitive Deficits in the Step-Through Passive Avoidance Task

**Figure 6:**
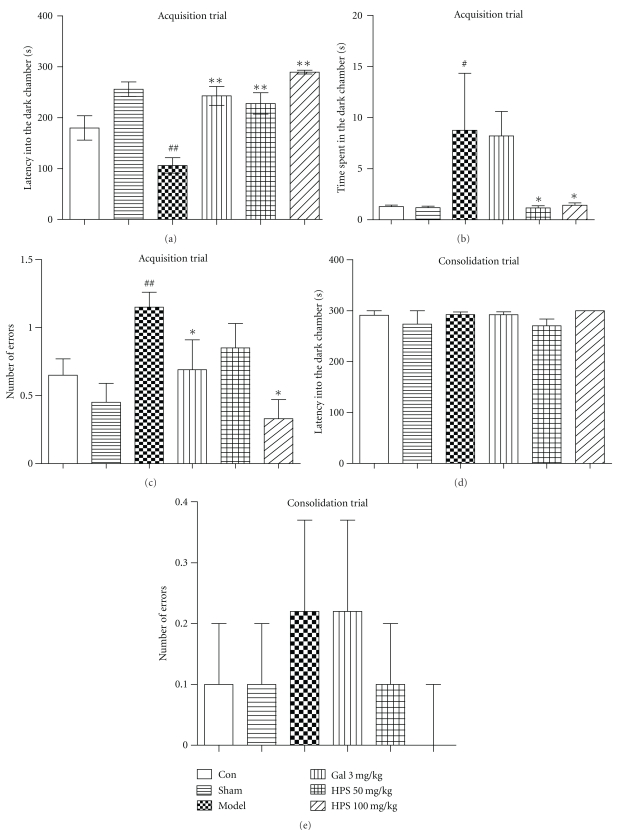
Effect of HPS on A*β*_25–35_-induced memory deficits in the step-through passive avoidance test in mice. (a) Latency into the dark chamber, (b) time spent in the dark chamber, and (c) number of errors were detected in memory acquisition stage. 24 h later, (d) latency into the dark chamber, and (e) Number of errors were observed to assess the effect of HPS on memory consolidation. Values represent mean ± S.E.M (*n* = 8–10 in each group). Gal 3 mg/kg represents the galantamine 3 mg/kg group. ^#^ *P* < .05, ^##^ *P* < .01, versus sham-operation group. *P < .05; **P < .01, versus model group.

One-way ANOVAs revealed a significant group effect on the acquisition trial of the step-through test for the latency into the dark chamber (*F*_5,50_ = 12.51, *P* < .001, Figure 6(a)), the time spent in the dark chamber (*F*_5,50_ = 2.46, *P* = .050, Figure 6(b)), and the number of errors (*F*_5,50_ = 3.07, *P* = .017, Figure 6(c)). LSD *post hoc* comparison showed that the latency into the dark chamber was significantly shorter in A*β*_25–35_-injected mice than that of sham-operated mice (*P* < .001). Galantamine (3 mg/kg) increased the latency time significantly (*P* < .001), and HPS (50 and 100 mg/kg) prolonged the latency time significantly (*P* < .001) in a dose-dependent manner. On the contrary, the time spent in the dark chamber (Figure 6(b)) and the number of errors (Figure 6(c)) were increased markedly in the model group compared to sham group (*P* = .028 and  .003, resp.). HPS (50 and 100 mg/kg) could decrease the time spent in dark chamber significantly (*P* = .028 and *P* = .044). HPS (100 mg/kg) and galantamine (3 mg/kg) also reduced the number of errors significantly (*P* = .002 and  .050, resp.). However, in the consolidation trial (Figures 6(d) and 6(e)), no significant differences were observed in all groups (Kruskal Wallis test, latency into the dark chamber: df = 5, *P* = .558; number of errors: df = 5, *P* = .466).

### 3.4. Effect of HPS on SOD Activities and MDA Levels in Cortex and Hippocampus

**Figure 7:**
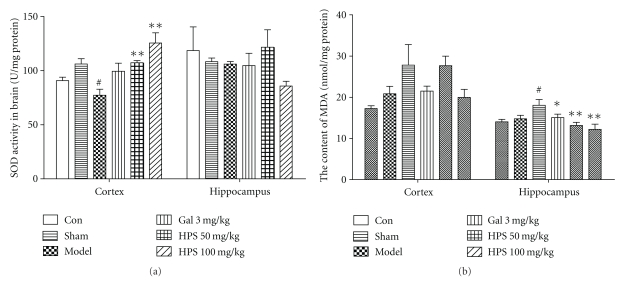
Effect of HPS on the changes of SOD activities (a) and MDA levels (b) induced by A*β*_25–35_ in the cortex and hippocampus of mice. Each value represents the mean ± S.E.M (*n* = 8–10 in each group). Gal 3 mg/kg represents the galantamine 3 mg/kg group. ^#^ *P* < .05,  ^##^ *P* < .01, versus sham-operation group. *P < .05; **P < .01, versus model group.

The difference of SOD activities among groups was significant in the cortex (*F*_5,51_ = 5.741 = 6.967,  *P* < .001) but not in the hippocampus (*F*_5,51_ = 1.072, *P* = .387). As shown in Figure 7(a), the SOD activities of the cortex decreased significantly in the model group compared with the sham group (by 25.72%, *P* = .014). Mice in the HPS (50 and 100 mg/kg) and the galantamine (3 mg/kg) group showed higher SOD activities than mice in the model group (by 34.47%, *P* = .005; 63.34%, *P* = .006; 43.25%, *P* = .219, resp.). However, there was only a slight increase of SOD activities in the hippocampus of the HPS group (50 mg/kg), and no difference was observed compared with model group (by 14.78%, *P* > .05). On the contrary, the MDA levels among groups were significantly different in the hippocampus (*F*_5,49_ = 4.225, *P* = .003) but not in the cortex (*F*_5,51_ = 2.852,  *P* = .233), as shown in Figure 7(b). The MDA levels of A*β*_25–35_-injected mice were significantly higher than those of sham-operated mice in the hippocampus (by 23.81%, *P* = .013), and had the same tendency in the cortex (by 22.21%, *P* > .05). Galantamine (3 mg/kg) and HPS (50 and 100 mg/kg) decreased the MDA levels in the hippocampus significantly (by 16.57%, *P* = .027; 27.26%, *P* = .001; 32.35%, *P* < .001, resp.), and in the cortex with a similar tendency (by 19.62%, 8.10%, and 22.57%, resp.) without statistical difference.

## 4. Discussion

It is well known that A*β* deposit-induced neurotoxicity is widely regarded as having a pivotal role in the development of AD [33]. Cognitive deficit has been well documented in rodents with a centrally administered A*β* synthetic peptide, such as A*β*_1–40_ [24, 31, 33–35] or A*β*_1–42_ [36] analogous to peptides found in neuritic plaques in AD patients. The peptide fragment A*β*_25–35_, a subset of A*β*_1–42_ located at the C-terminal end in the hydrophobic domain, with only 11 amino acids, was also reported to be responsible for the neurotoxic effect of A*β* [37, 38]. In the present study, a single intrahippocampal injection of A*β*_25–35_ in C57BL/6J mice (6 *μ*g/mouse) did result in deficits in learning and memory. Furthermore, these behavior deficits were accompanied by a decrease of SOD activities and an increase of MDA levels in the cortex and hippocampus, which is in accordance with previous studies showing that A*β*-induced cytotoxicity is associated with free radical oxidative stress which results in the formation of reactive oxygen species, lipid peroxidation, and modification of proteins by reactive lipid peroxidation products in neurons [39–41]. These changes are similar, at least in part, to features of the AD process. Therefore, the A*β*_25–35_-injected animal model has been widely used to evaluate the effect of anti-amnesia agents. Accordingly, deficits produced by selective lesions of the hippocampus can be detected by hippocampally dependent learning tasks, such as Morris water maze [29] and inhibitory avoidance [42–44]. 

In our preliminary study, we found that polygalasaponins were effective in improving learning and memory but were also toxic to animals, leading to nose bleeding, gastrointestinal tracts abnormality (hyperemia, gaseous distention, and necrosis), and even death. Acute toxicity experiments showed that HPS, a hydrolysate of polygalasaponins, decreased the toxicities markedly (data not shown). The present findings demonstrate for the first time that HPS clearly improves cognitive deficits induced by intrahippocampal injection of aged A*β*_25–35_ in mice as observed in Morris water maze and step-through passive avoidance tests. Furthermore, the antiamnesic effect observed here may be mediated by its antioxidant activities. 

It is important to notice that AD is associated with a variety of hippocampus-dependent memory deficits, such as a defect in long-term memory formation [45]. Morris water maze is generally accepted as an indicator of spatial learning and reference memory, which reflects long-term memory [29, 46]. Spatial learning is assessed across repeated trials and reference memory is determined by preference for the platform area when the platform is absent [46]. In the present study, we found that, between 10 and 13 days after intrahippocampal injection of A*β*_25–35_, mice in the model group showed significant long-term memory impairments. The results are similar to previous studies which demonstrated that water-maze place learning and probe trial performance were impaired 10–14 days after i.c.v. injection of A*β*_25–35_ in mice [37]. The HPS (100 mg/kg) significantly shortened the escape latency prolonged by A*β*_25–35_ injection after 2 days of training. Interestingly, when using the escape rate as an index of learning and memory, HPS showed strong improving effects on all 3 days. HPS (100 mg/kg) increased the escape rate to approximately 63%, 91%, and 100% on 3 days of training, compared to that in the model group (32%, 69%, and 84%). In addition, during the probe trial session, HPS (50 and 100 mg/kg) increased the swimming time and distance in the quadrant where the platform was previously placed up to 37.44% and 37.59%, respectively, as did the galantamine-treated group. This effect was not due to a different motor activity, because the mean swimming speed is not significantly different among all groups. Moreover, the model group and HPS-treated groups showed comparable locomotor activity and emotional reactivity in the open-field test, which can also suggest that the amelioration was not provoked by sensorimotor effects (Figure 2). Taken together, it is likely that HPS ameliorates the spatial reference and long-term memory in the amnesic model induced by A*β*_25–35_ intrahippocampal injection. Apart from that, an interesting observation emerged from the results in the Morris water maze, suggesting that different indexes had different sensitivities to evaluate the learning and memory abilities. Multiple index analysis may be more effective and accurate. 

To confirm the effects of HPS on other types of learning and memory, the step-through passive avoidance task, a fear-motivated inhibitory avoidance test indicating nonspatial learning and memory, was performed on days 15 and 16 after surgery. The results showed that in the training trial, latencies into the dark chamber did not vary among all groups, indicating no difference in exploratory activity and motor behavior induced by the treatment (data not shown). A*β*_25–35_ impaired the acquisition but not the retention of avoidance behavior in the step-through test. Giovannelli et al. [24] similarly demonstrated memory impairments in the step-through test in the first two weeks after intracerebral injection of A*β*_25–35_, but the impairments were observed in the retention trial and disappeared 21 days after surgery. This discrepancy may be related to the use of one-trial learning protocol instead of the multitrial method we used. Treatment with HPS (50 and 100 mg/kg) could ameliorate the memory deficits significantly in the acquisition trial (Figure 5). Besides, evidence from the inhibitory avoidance test indicated that the aversive conditioning is learned rapidly and fear memories tend to be very stable [44]. Gale et al. [47] have observed that the fear response such as robust freezing to contex lasted for 16 months following fear conditioning. In the present study, 24 h after foot shock, mice in all groups showed freezing and scrunching at the place away from the dark chamber, and no difference was observed in all groups. 

Galantamine, a plant alkaloid, is an acetylcholinesterase inhibitor most commonly used to treat AD in clinics. However, in the present study, galantamine (3 mg/kg, p.o.) showed only a slight ameliorative effect on spatial cognition in Morris water maze, which was weaker than HPS treatment. In addition, the exploration and locomotion activity was significantly decreased in the galantamine group. It is congruent with previous reports that galantamine (1.25–5.0 mg/kg, i.p.) is effective in improving cognition in several behavior tests in rodent models exhibiting cognitive deficits [48–51], as motor activity was severely reduced [52]. Interestingly, galantamine (3 mg/kg, p.o.) had a significant improvement effect on passive avoidance behavior in the step-through task. A potential alternative explanation of these discrepant results in two behavior tasks may be a difference in sensitivities of the two behavior tasks and/or difference in the effect of galantamine on learning and memory. Clinical data have shown that galantamine improves cognitive function and behaviour in patients who have mild to moderate AD, but has no effect on activities of daily life of patients with AD [53]. The present results obtained with galantamine also indicate a limitation of acetylcholinesterase inhibitors with regard to therapeutic usefulness for AD patients. 

Although the exact mechanisms responsible for the cognitive impairment induced by A*β*_25–35_ administration in mice are still unclear, evidence has accumulated that it may be related to oxidative stress [39–41, 54]. Oxidative stress occurs when pro-oxidant and antioxidant levels become imbalanced. SOD, as an endogenous antioxidant enzyme, plays an important role in the intracellular antioxidant defense in the brain. In the current study, A*β*_25–35_ administration led to a significant decrease of SOD activities in the cortex (by 25.72%) but had no marked changes in the hippocampus (by 3.66%). HPS (100 mg/kg) increased the SOD activities in the cortex significantly (by 62.34%), and to a lesser extent (22.09%) in the hippocampus, which suggests that HPS partially reversed the antioxidant capacity impaired by A*β*_25–35_. Decreased activities of SOD further promote the accumulation of free radicals and/or reactive oxygen species (ROS), which initiate the oxidative damage downstream and cause lipid peroxidation in the brain [55–57]. MDA, an important lipid peroxidation product, can be taken as an indicator for the state of oxidative damage of membranes under conditions of oxidative stress. The reported abnormal alteration in MDA content and its relation to memory impairment have been shown in previous studies [58]. We found that HPS (50 and 100 mg/kg) decreased the MDA levels of the hippocampus significantly, but did not change that in the cortex obviously. The relationship between SOD activities and MDA levels is somewhat controversial. The difference may be related to other types of endogenous antioxidants such as glutathione and GSH-Px [40], and maybe they also exert the antioxidant effect simultaneously, which needs to be further investigated. 

In conclusion, the findings described in the present study provide important information on the improvement effect of HPS on learning and memory in an intrahippocampal-injected A*β*_25–35_ mouse model. HPS not only has an antiamnesic effect, but it also decreases the toxicities of polygalasaponins, and may be an effective agent for AD treatment, via antagonizing oxidative damage induced by A*β*_25–35_. 

##  Funding

This paper was supported by grants from the International Science and Technology Cooperation Program of China (2009R0002) and the National Science and Technology major projects (2009ZX09502-014, 2009ZX09301-003-15-03).
